# MCCC2 promotes HCC development by supporting leucine oncogenic function

**DOI:** 10.1186/s12935-020-01722-w

**Published:** 2021-01-06

**Authors:** Yu-Yan Chen, Xue-Ning Zhang, Chen-Zhou Xu, Dan-Hua Zhou, Jing Chen, Zhao-Xiu Liu, Ying sun, Wei Huang, Li-Shuai Qu

**Affiliations:** 1 Department of Gastrointestinal Surgery, Affiliated Hospital of Nantong University, Nantong, China; 2 Research Center of Clinical Medicine, Nantong University, Affiliated Hospital of Nantong University, Nantong, China; 3 Department of Gastroenterology, Affiliated Hospital of Nantong University, Nantong, China; 4 Department of Gastroenterology, The First Hospital of Jiaxing, Affiliated Hospital of Jiaxing University, Jiaxing, China; 5 Blood Center of Jiangsu Province, Nanjing, China

**Keywords:** Hepatocellular carcinoma, MCCC2, leucine, ERK, metabolism

## Abstract

**Background:**

The role of methylcrotonoyl-CoA carboxylase 2 (MCCC2) in the development of tumors is well-established, and the involvement of leucine in the liver is well-known. However, the role of MCCC2 and the correlation between MCCC2 and leucine in the progression of hepatocellular carcinoma (HCC) have not yet been reported.

**Methods:**

In this study, the Gepia database was used to evaluate the prognostic value of MCCC2 in HCC. The expression and localization of MCCC2 in HCC cells were determined by western blot and immunofluorescence assays. Flow cytometry and CCK-8 and transwell assays were carried out to explore the effect of MCCC2 on cell proliferation, migration, and invasion. In addition, mass spectrometry analysis was used to predict the potential cell function of MCCC2 in HCC.

**Results:**

We found that the expression of MCCC2 increased in HCC tissues and that high expression of MCCC2 could predict poor outcomes in HCC patients. Knockdown expression of MCCC2 in HCC cells could reduce cell proliferation, migration, and invasion ability in vitro and could inhibit HCC cell proliferation in vivo. Interestingly, we found that HCC cells transfected with MCCC2-sgRNA failed to respond to leucine deprivation. Meanwhile, leucine deprivation inhibited cell proliferation, migration, and invasion in HCC cells where MCCC2 was present rather than in cells where MCCC2 was absent. In addition, knockdown of MCCC2 significantly reduced the glycolysis markers, glucose consumption, lactate secretion, and acetyl-CoA level, which is a product of leucine metabolism. Furthermore, we found that MCCC2 promotes the activation of ERK. Profiling the MCCC2 binding proteins revealed that MCCC2-associated proteins are enriched in biological processes, such as protein metabolism, energy pathway, and metabolism in HCC cells.

**Conclusions:**

Our findings revealed that MCCC2 plays a critical role in the development of HCC, and the leucine metabolism pathway might be a novel target in HCC treatment.

## Introduction

Tumors are one of the major causes of death worldwide and have become an important factor hindering the improvement of life expectancy. The incidence and mortality of liver cancer worldwide ranked seventh and second for all cancers, respectively, in 2018, accounting for 4.7% and 8.2% of all tumors, respectively. Hepatocellular carcinoma (HCC) is the most prevalent type of liver cancer, accounting for 90% of all liver cancer cases [1], and has a poor prognosis and a high recurrence rate. However, the molecular mechanisms underlying the development of HCC remain largely unclear [2]. Therefore, there is an urgent need to decipher the underlying mechanisms of HCC, which may provide a novel diagnostic and therapeutic strategy for HCC patients.

A major abnormality of cancer cells is a change in their metabolism [3]. Cancer cells can change their metabolism and obtain the necessary nutrients from an undernourished environment, gain energy, and build new biomass [4, 5]. Abnormal metabolism of cancer is mainly characterized by upregulation of glucose and amino acid uptake, changes in metabolite-driven gene regulation, interaction with the microenvironment, etc. [6]. LLGL2 can promote the proliferation of tumor cells by accelerating leucine uptake in ER^+^ breast cancer [7]. As one of the branched chain amino acids, leucine can avoid first-pass liver catabolism in the human body to transport nitrogen throughout the body for the synthesis of non-essential amino acids, such as the neurotransmitter glutamate [8]. In some malignant tumors, the growth of tumor cells is also closely related to the catabolism of glutamine [9, 10]. In addition, there is direct evidence that the inhibition of BCAT1 in glioma cell lines blocks glutamate excretion, leading to reduced proliferation and invasiveness in vitro [11]. These data indicate that different cancers might require particular amino acids for survival because of their distinct oncogenic transformation. However, the role of leucine in HCC is currently unknown.

3-methylcrotonyl-CoA carboxylase (MCC), a member of the biotin-dependent carboxylase superfamily [12], has a high degree of conservation of PCC. The entire enzymatic structure is a heterododecyl, consisting of six alpha and six beta subunits [13]. MCCase was first discovered in bacteria and mammals 48 years ago [14], and it can decompose leucine into acetoacetate and acetylCoA [15, 16]. By interacting with SIRT4 [17], the activity of MCC increases, thereby promoting the catabolism of leucine [18]. As a subunit of MCC, methylcrotonoyl-CoA carboxylase 2 (MCCC2) is highly expressed in breast and prostate cancers and can promote the proliferation and metastasis of tumor cells [19–21]. For example, the high expression of MCCC2 predicts a poor prognosis and can promote cell proliferation in colorectal and breast cancer [19, 22]. The oncogenic role of MCCC2 is partially involved in the GLUD1-P38 MAPK signaling pathway [23]. However, it is currently unclear whether MCCC2 also plays an important role in the progression of HCC and the effect of MCCC2 on leucine metabolism has yet not been deciphered.

In this study, we found that MCCC2 participates in the utilization of leucine by HCC cells, thereby promoting the proliferation, migration, and invasion of HCC cells.

## Materials and methods

### Cell culture and cell lines

Human HCC lines (SMCC-7721, SK-HEP1, HepG2, and Huh7) and the normal liver cell line LO2 were obtained from the Shanghai Institute of Cell Biology, Academic Sinica. The cells were cultured in RPMI 1640 and Dulbecco’s modified Eagle’s medium (DMEM) (Hyclone, USA; Caisson, USA, Lot:04191017), respectively, supplemented with 10% (vol/vol) fetal bovine serum (Gibco, USA) 10 000 U/mL penicillin, and 10 000 µg/mL streptomycin (Gibco, USA) in 5% CO_2_ at 37 °C.

### sgRNA construction and stable cell line establishment

The sgRNAs targeting MCCC2 were a gift from Dr. Li at the Houston Methodist hospital [24]. To establish stable cell lines, SMMC-7721 cells were transfected with the plasmids then screened with 1 µg/mL of puromycin (ApexBio, Houston, TX, USA), and stable expression cell lines were obtained. The protocols were carried out as described previously [25]. The cloned sgRNA sequences were as follows:

SgMCCC2-1: GTGCCCGCGCCTCTCCCGCC;

SgMCCC2-2: GCGCGCCTATCACGGGGACT;

SgMCCC2-3: GGCCTCGCTGGGCACCCAGC.

### Western blot

Cells were lysed in RIPA lysis buffer (50 mmol/L Tris pH 7.4, 120 mmol/L NaCl, 1% Triton X-100, 1% sodium deoxycholate, 0.1% SDS, Beyotime, Shanghai, China) supplemented with protease inhibitors (Complete Mini, Roche, Basel, Switzerland) and phosphatase inhibitors (PhosSTOP, Roche).

Primary antibodies: MCCC2 (12117-1-AP; Proteintech, Rosemont, IL, USA), PCNA (sc-56, Santa Cruz), c-Myc (#D84C12; CST, USA), E-cadherin (#14,472; CST), N-cadherin (#13,116; CST), MMP9 (#13,667; CST), phospho-p44/42 MAPK (Erk1/2) (#4370; CST), p44/42 MAPK (Erk1/2) (#9102; CST), phospho-Mek1 (Thr286) (#9127; CST), Flag (ab002-100; Multi Sciences, Hangzhou, China), and GAPDH (60004-1-Ig; Proteintech, Rosemont, IL, USA).

### Flow cytometry

Cells were fixed with 70% ethanol at − 20 °C overnight and washed three times with cold PBS. The samples were stained with PI/RNase Staining Buffer (BD Pharmingen, Franklin Lakes, NJ, USA) for 15 min at room temperature according to the manufacturer’s instructions. Stained cells were analyzed with a BD FACSCanto II Flow Cytometer. The results were analyzed using ModFit LT 3.1.

### Nuclear protein and cytoplasmic protein extraction

After the cells were washed with PBS, they were scraped off with a cell scraper and collected by centrifugation to leave a cell pellet. After extraction of the nucleus and cytoplasm using a Nuclear and Cytoplasmic Protein Extraction Kit (Beyotime Biotechnology, Wuhan, China) western blotting was performed.

### LC-MS/MS identification of proteins

Cells were lysed in RIPA Lysis Buffer (50 mM Tris pH 7.4, 120 mM NaCl, 1% Triton X-100, 1% sodium deoxycholate, 0.1% SDS, Beyotime, China) supplemented with protease inhibitors (Complete Mini, Roche, Switzerland) and phosphatase inhibitors (PhosSTOP, Roche, Switzerland). Then, 1000 µg of total cell lysates were incubated with 1 µg of primary antibody or control rabbit IgG at 4 ℃ overnight. Then, 20 µL of Protein A + G agarose (Bioworld Technology, USA) was added for 2 h at 4 °C with rocking. The precipitates were washed four times with RIPA lysis buffer (50 mM Tris pH 7.4, 150 mM NaCl, 1% NP-40, 0.5% sodium deoxycholate, 0.1% SDS, Beyotime, China).

Analysis was conducted on a Q Exactive mass spectrometer coupled to an Easy nLC (Thermo Fisher Scientific, USA) using a routine method. MS data were acquired using a data-dependent top 10 method dynamically selecting the most abundant precursor ions from the survey scan (300–1800 m/z) for HCD fragmentation. The proteins identified from the blank control group were regarded as non-specific proteins and were removed from the protein list identified from the MCCC2 test group to exclude the non-specifically binding proteins of MCCC2.

### Wound healing assay

Cells were seeded into six-well plates and transfected as previously described. After the cells were cultured to 90%, a line was gently etched on the bottom with a 100 µL perpendicular tip. The lines were made as straight as possible. Excess cellular debris was removed with cell PBS (Hyclone, USA). Then, serum-free medium was added to incubate the cells at 37 °C. The lines were photographed using a Nikon confocal microscope (Nikon, NY, USA) at 0, 24, and 48 h.

### Transwell assays

Transwell assays were conducted in 24-well plates to investigate the invasiveness of the tumor cells. Cells were resuspended in serum-free medium and seeded into the upper chamber. There were approximately 1 × 10^5^ cells in each upper chamber. According to the manufacturer’s instructions, we prepared and used Matrigel. The bottom chamber was filled with 500 µL of medium containing 10% FBS. The cells were then incubated at 37 °C for 24 h. After incubation in xylene for 1 h and staining with crystal violet. The cells were wiped inside the room with a cotton swab. A microscope was used to observe and take pictures.

### Xenograft mouse model

Nude mice were obtained from the Nantong University Laboratory Animal Center. SMMC-7721 cell suspension in DMEM (5 × 10^6^/mouse, n = 3) was subcutaneously injected into the male nude mice. Mice were euthanized eight weeks after implantation, and tumor xenografts were collected.

### Immunocytofluorescence (IF)

Cells were fixed with 4% paraformaldehyde in PBS for 1 h at room temperature then permeabilized with 1% Triton X-100 in PBS for 20 min. The cells were blocked with 1% BSA in PBS for 1 h and incubated with primary antibodies overnight at 4 °C. Secondary antibody incubation was performed using Alexa Fluor 568 donkey anti-mouse or Alexa Fluor 488 donkey anti-rabbit antibodies (Invitrogen, Carlsbad, CA, USA) for 1 h at room temperature. Then, 5 mg/mL DAPI was added to stain the nucleus. The slides were mounted and visualized using a Nikon confocal microscope (Nikon, Melville, NY, USA).

### Lactate and glucose measurements

Briefly, glucose concentrations in the media were determined using a glucose colorimetric assay kit (BioVision, Milpitas, CA, USA) and lactate secretion was determined using a lactate colorimetric assay kit (BioVision) following the manufacturer’s instructions.

### Total RNA extraction and quantitative real-time polymerase chain reaction (RT-qPCR)

RNA extraction and real-time RT-PCR were performed as previously described [7]. All primers were purchased from Sangon (Shanghai, China). Primer sequences were as follows: GLUT1 forward: 5′-CAGTTTGGCTACAACACTGGAG-3′; GLUT1 reverse, 5′-GCCCCCAACAGAAAAGATGG-3′; LDHA forward: 5′- TGGAGATTCCAGTGTGCCTGTATGG-3′; LDHA reverse: 5′- CACCTCATAAGSACTCTCAACCACC-3′; LDHB forward: 5′-GGAAGGAAGTGCATAAGATGGTGG-3′; LDHB reverse: 5′-CCCCTTTACCATTGTTGACACG-3′; GAPDH forward: 5′-AGAAGGCTGGGGCTCATTTG-3′; GAPDH reverse: 5′-AGGGGCCATCCACAGTCTTC-3′.

### Acetyl-CoA ratio measurement assay

Briefly, AcCoA content was determined from total or cytosolic fractions using the acetyl-CoA assay kit (ab87546, Abcam) according to the manufacturer’s instructions.

### Statistical analysis

All data were analyzed and displayed using GraphPad 8.0.2 (GraphPad Software, Inc., La Jolla, CA, USA). Student’s t-test analysis was used to analyze differences between groups. The Kaplan-Meier method was used to analyze the survival curves. For all tests, a P-value of less than 0.05 was considered statistically significant.

## Results

### MCCC2 is upregulated in HCC and predicts a poor prognosis for HCC patients

**Fig. 1:**
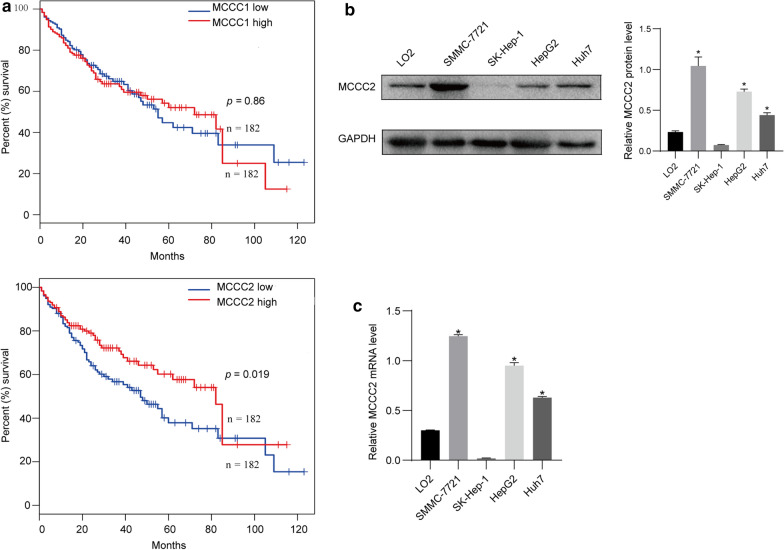
The expression of MCCC2 is upregulated and predicts a poor prognosis in HCC. **a** TCGA database were used to analyze the relationship between the expression of MCCC1 and MCCC2 and the survival rate in HCC patients. **b** Western blot images demonstrated the expression of MCCC2 in HCC cell lines and the normal liver cells. **c** The expression of MCCC2 in HCC cell lines and the normal liver cells was detected by qRT-PCR

Although MCCC1 and MCCC2 are involved in the catabolism of leucine, their roles in the development of HCC are largely unknown. In this study, TCGA dataset with an online program (https://www.gepia.cancer-pku.cn) [26] was used to explore the correlation between HCC patient survival and the expression of MCCC1 and MCCC2. Interestingly, we found that high expression of MCCC2 but not MCCC1 predicts a poor prognosis in HCC patients (Fig. 1a). Next, we examined the expression of MCCC2 in cell lines by western blotting. Compared to normal liver LO2 cells, the expression of MCCC2 in SMMC-7721 and HepG2 was largely increased at the protein level (Fig. 1b). However, the expression of MCCC2 in SK-HEP-1 cells was undetectable. Similar results were observed at the RNA level (Fig. 1c). Collectively, these results suggest that MCCC2 may play an important role in the progression of HCC.

### MCCC2 regulates HCC cell proliferation, migration, and glycolysis ***in vitro***

**Fig. 2:**
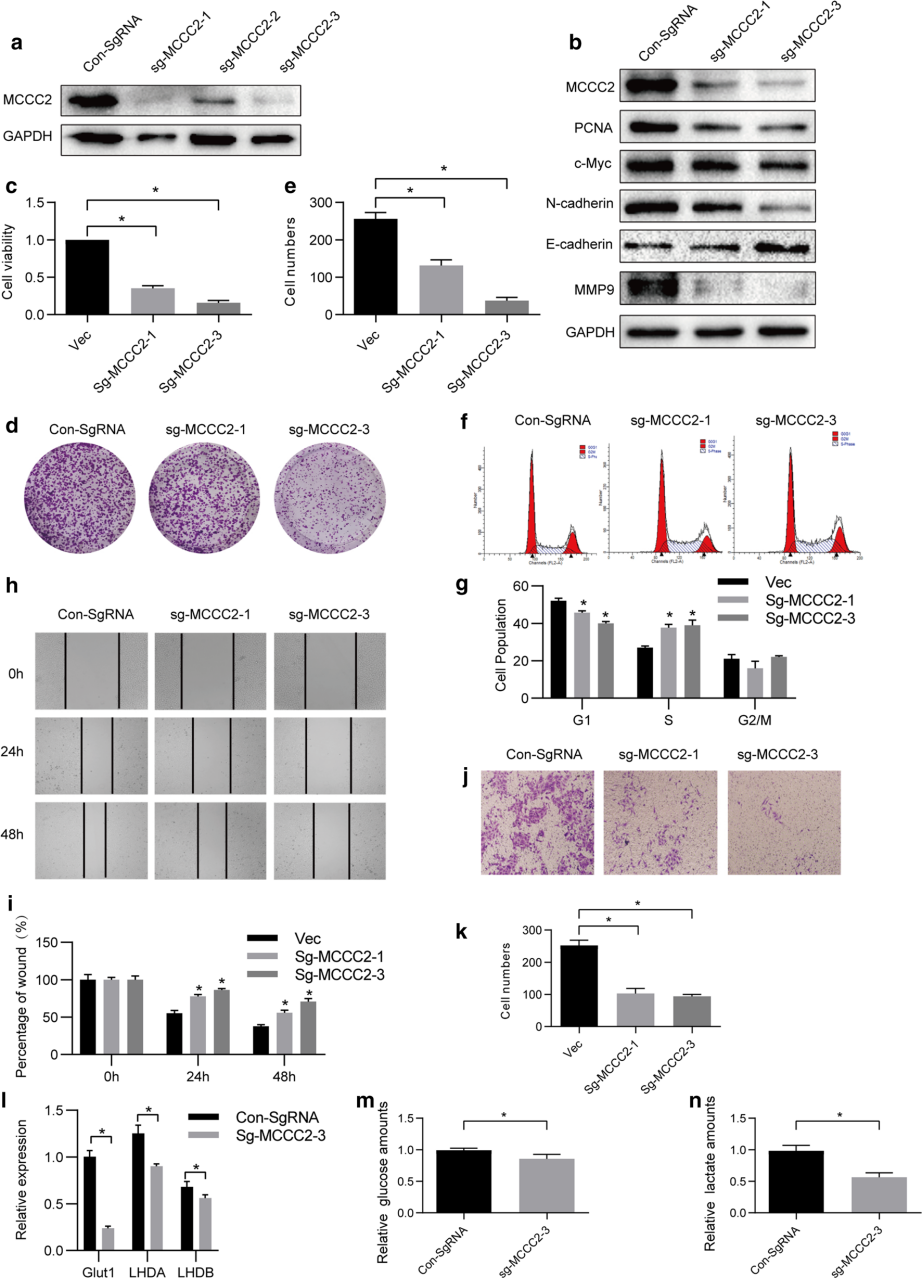
MCCC2 promotes the proliferation, migration, and invasion of HCC cells. **a** The expression of MCCC2 was determined using western blot after transfection. **b** Western blot analysis was used to detect the relative protein levels of MCCC2, PCNA, c-Myc, N-cadherin, N-cadherin, and MMP9 in the MCCC2 low expression group and control group for SMMC-7721 cell lines. **c** SMMC-7721 cells were transfected with vector sg-MCCC2 to examine the proliferation of SMMC-7721 cells by CCK-8 assay. **d**, **e** The cells were transfected and seeded into six-well plates for 2W. **f**, **g** Flow cytometry was performed to determine the cell cycle distribution of the cells that were transfected. The cell cycle distribution is displayed in the bar chart. **h**, **i** Wound-healing assay. HCC cells were transfected with sg-MCCC2 and vector. The wound was observed at 0, 24, and 48 h under a light microscope. **j**, **k** Transwell assay. Cells transfected with sg-MCCC2 and vector travelled membrane were observed at 48 h. **l** qrt-PCR was used to detect Glut1, LHDA, and LHDB expression in the MCCC2 low expression and control groups for SMMC-7721 cell lines. **m**, **n** Glucose and lactate consumption was detected in the same groups

Next, we explored the role of MCCC2 in the biological behavior of HCC cells. Based on the expression of MCCC2 in HCC cell lines, SMMC-7721 cells were transfected with sgRNA and the stable cell line was successfully established (Fig. 2a). As shown in Fig. 2a, sg-MCCC2-1 and MCCC2-2 with the best knockdown effect were selected for subsequent experiments. Next, we detected the expression of tumor markers in SMMC-7721 cells after knocking down MCCC2. We found that the expressions of tumor proliferation-related markers PCNA and C-myc were reduced in SMMC-7721 cells transfected with sg-MCCC2-1 and sg-MCCC2-3 and found that the expression of N-cadherin, a mesenchymal cell-specific marker, decreased. However, knockdown of MCCC2 could upregulate the expression of E-cadherin, an epithelial cell marker. Simultaneously, we found that the invasion-related marker MMP9 expression was reduced in MMC-7721 cells transfected with sg-MCCC2-1 and sg-MCCC2-3 (Fig. 2b). These results suggest that MCCC2 plays an important role in cell metastasis and the invasion of HCC.

Interestingly, CCK-8 and clone formation experiments proved that knockdown of MCCC2 expression significantly inhibited cell proliferation compared with the control cells (Fig. 2c, e). Flow cytometric analysis revealed that SMMC-7721 cells transfected with sgRNA were arrested in the S phase (Fig. 2f, g). In addition, the scratch-wound healing assay and Transwell assay showed that knockdown of MCCC2 reduced the migration and invasion ability of SMMC-7721 cells (Fig. 2h, k). As described above, MCCase is involved in leucine metabolism, thus, we investigated whether MCCC2 regulates amino acid metabolism. As expected, the expression of Glut1, LHDA, and LHDB, which are glycolysis markers, decreased in SMMC-7721 cells transfected with sg-MCCC2-3 (Fig. 2l), and glucose consumption and lactate secretion also decreased (Fig. 2l–n). In summary, our results indicated that MCCC2 plays a critical role in cell proliferation, migration, invasion, and glycolysis in HCC cells in vitro.

### MCCC2 promotes HCC cell proliferation in vivo

**Fig. 3:**
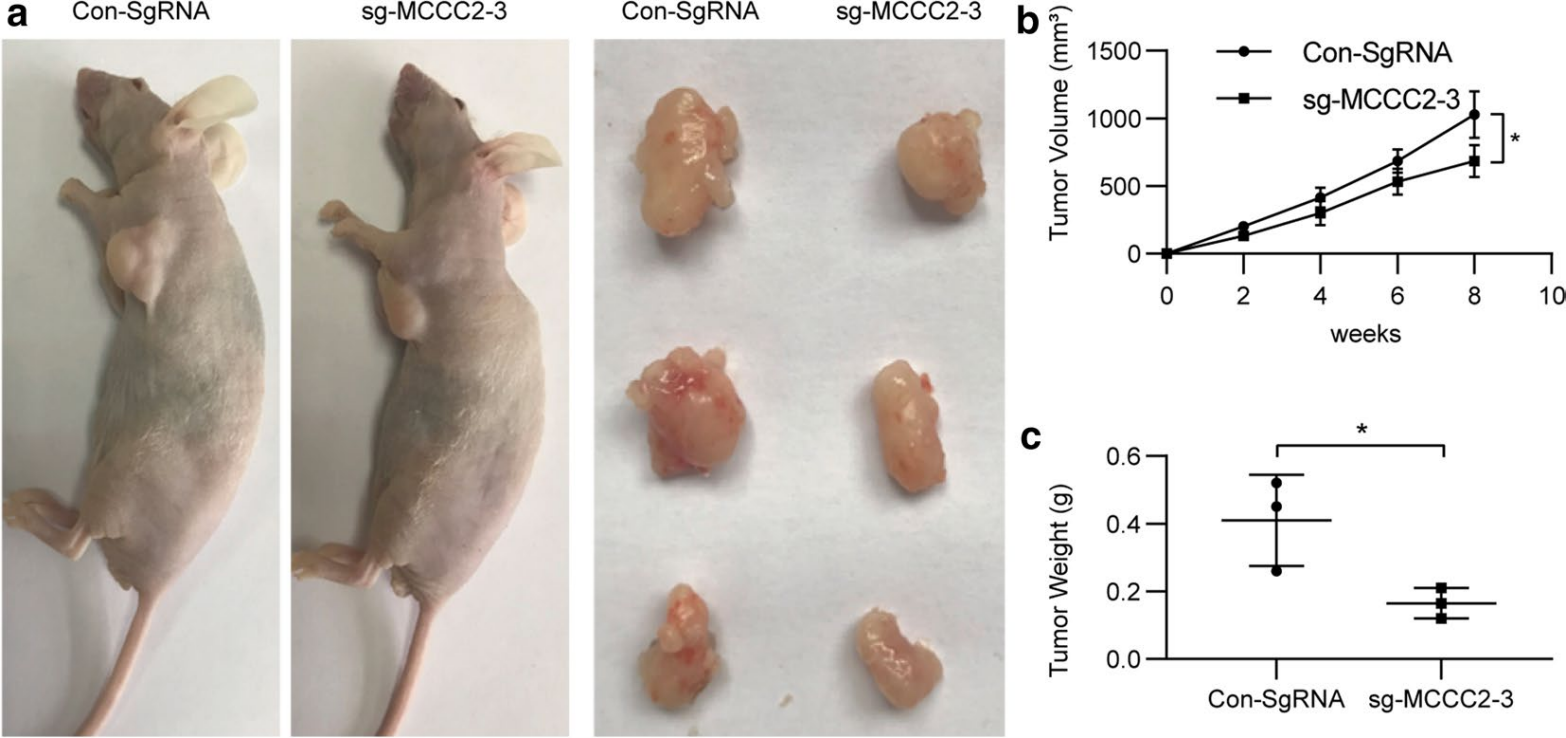
MCCC2 promotes HCC growth in vivo. **a** Xenograft tumors were generated by subcutaneously injecting SMMC-7721 cells knocking down MCCC2 or carrying a control vector. **b**, **c** The xenograft tumor weight was recorded. All *P < 0.05

To further explore whether knockdown of MCCC2 also affects the tumor formation of HCC cells in vivo, SMMC-7721 cells stably transfected with sg-MCCC2-3 or control vector were injected subcutaneously into nude mice. After six weeks, the subcutaneous tumors were smaller in the sg-MCCC2-3 group than in the control group (Fig. 3a, b). Moreover, the tumor weight in the sg-MCCC2-3 group was significantly lower than that in the control group (Fig. 3c). Therefore, our results demonstrate that knockdown of MCCC2 inhibits tumor growth in vivo.

### Cytoplasmic distribution of MCCC2 in HCC cells

**Fig. 4:**
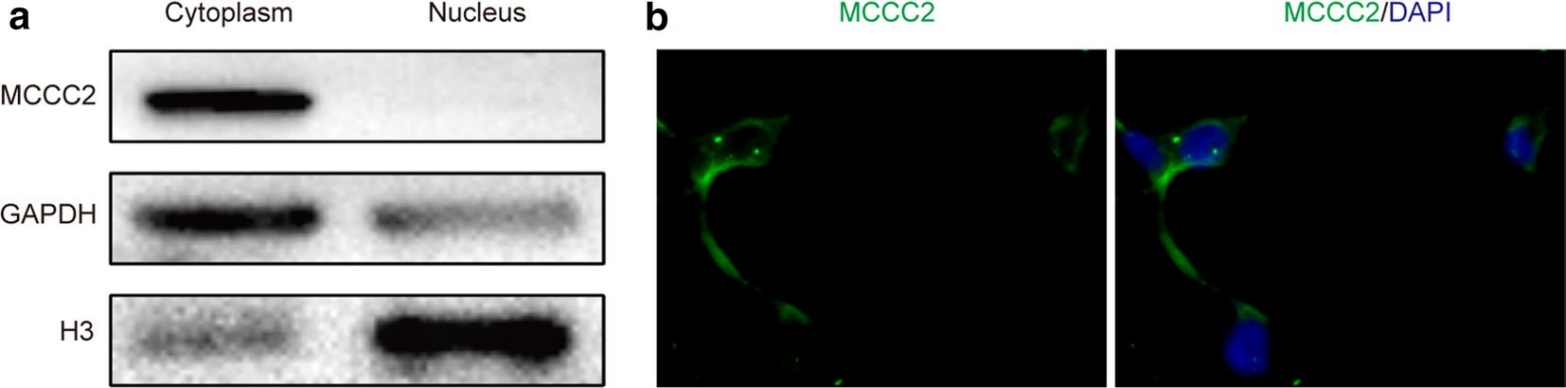
MCCC2 is distributed in the cytoplasm of HCC cells. **a** Western blot analysis of cytoplasmic and nuclear distribution of MCCC2. GAPDH and H3 in SMMC-7721 cell were used as specific markers for cytoplasmic and nuclear components, respectively. **b** Immunofluorescence analysis of MCCC2 in SMMC-7721 cells using anti-MCCC2 antibodies

To further explore the role of MCCC2, we examined the localization of MCCC2 in HCC cells. By separating the nuclear and cytoplasmic fractions of SMMC-7721 cells, we found that MCCC2 was mainly distributed in the cytoplasm (Fig. 4a). The cytoplasmic distribution of MCCC2 in HCC cells was further confirmed by immunofluorescence experiments (Fig. 4b). This is consistent with the localization of MCCC2 on mitochondria and its function in amino acid metabolism.

### The oncogenic function of MCCC2 in HCC cells is dependent on leucine

**Fig. 5:**
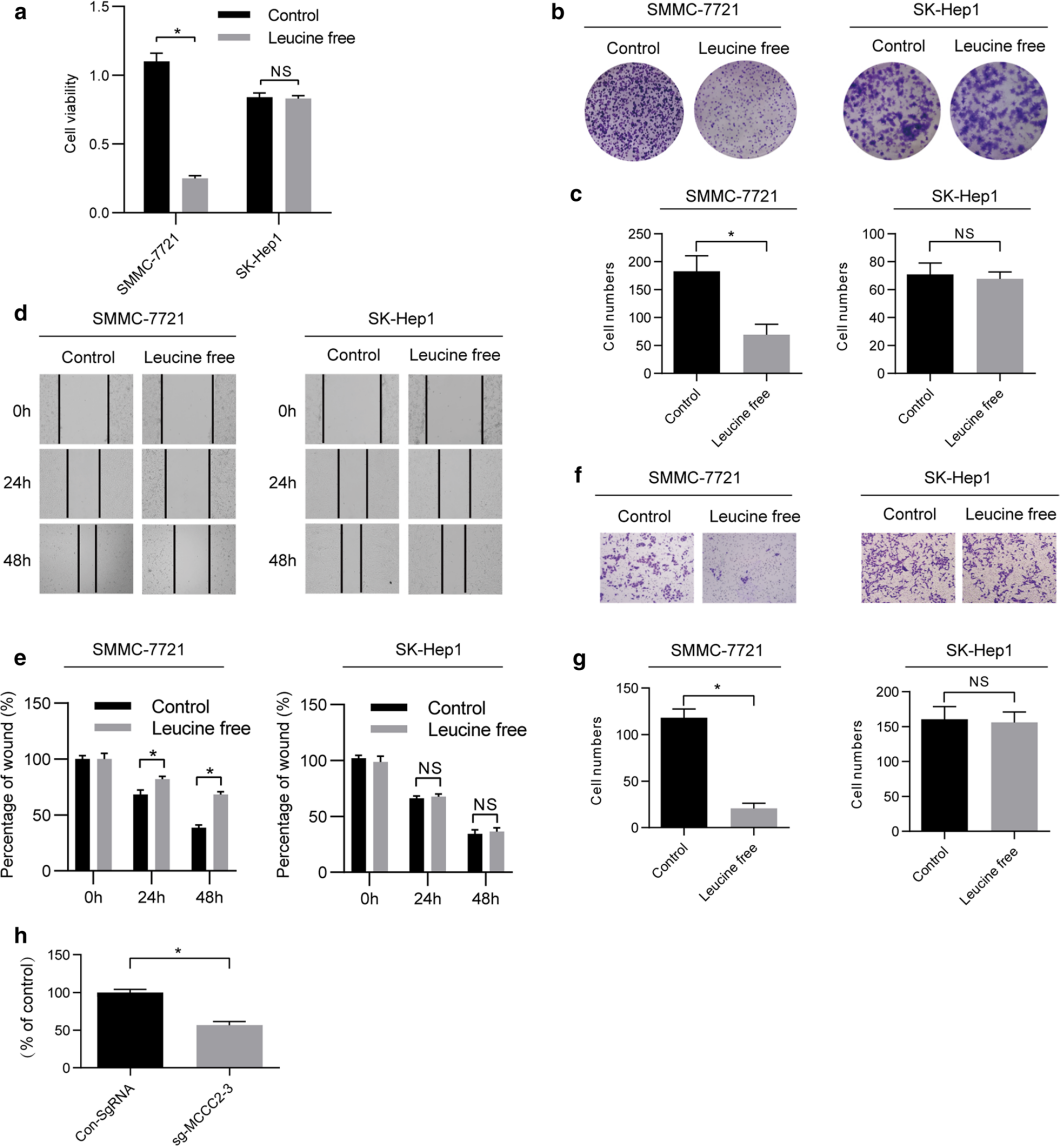
Absence of leucine fails to inhibit the biological characteristics of the HCC cells. **a** The following experiments were carried out in SMMC-7721 cell lines with relatively high MCCC2 expression and SK-Hep1 cell lines with no MCCC2 expression. The same number of cells were plated in 96-well plates and cultured under normal or leucine^−^ conditions, and the number and viability of cells were determined every day by CCK-8 assay. **b–g** Colony-formation, Wound-healing, and Transwell assays were performed with SMMC-7721 and SK-Hep1 cells treated as described above. **h** Acetyl-CoA levels were assessed with the knockdown of MCCC2 in SMMC-7721 cells

MCCC2 can affect leucine metabolism [27]. It is not currently known whether the oncogenic function of MCCC2 is also associated with leucine metabolism. To examine the effect of leucine on HCC cells, SMMC-7721 cells were cultured with leucine-free medium and it was found that the proliferation, migration, and invasion capabilities of SMMC-7721 cells were significantly reduced compared with the control cells (Fig. 5a, g). This suggested that SMMC-7721 cells were largely dependent on the presence of leucine in culture. Interestingly, we found that SK-HEP-1 cells, which lack the expression of MCCC2 (Fig. 1b, c), failed to respond to leucine deprivation. MCCCs can decompose leucine into acetyl-CoA [27], which is the final product of leucine metabolism. Meanwhile, the level of acetyl-CoA significantly decreased in SMMC-7721 cells transfected with sg-MCCC2-3 compared to in control cells (Fig. 5h). These results suggest that HCC cells with the expression of MCCC2 are dependent on leucine.

### Leucine deprivation failed to inhibit HCC cell growth in MCCC2 knockdown cells

**Fig. 6:**
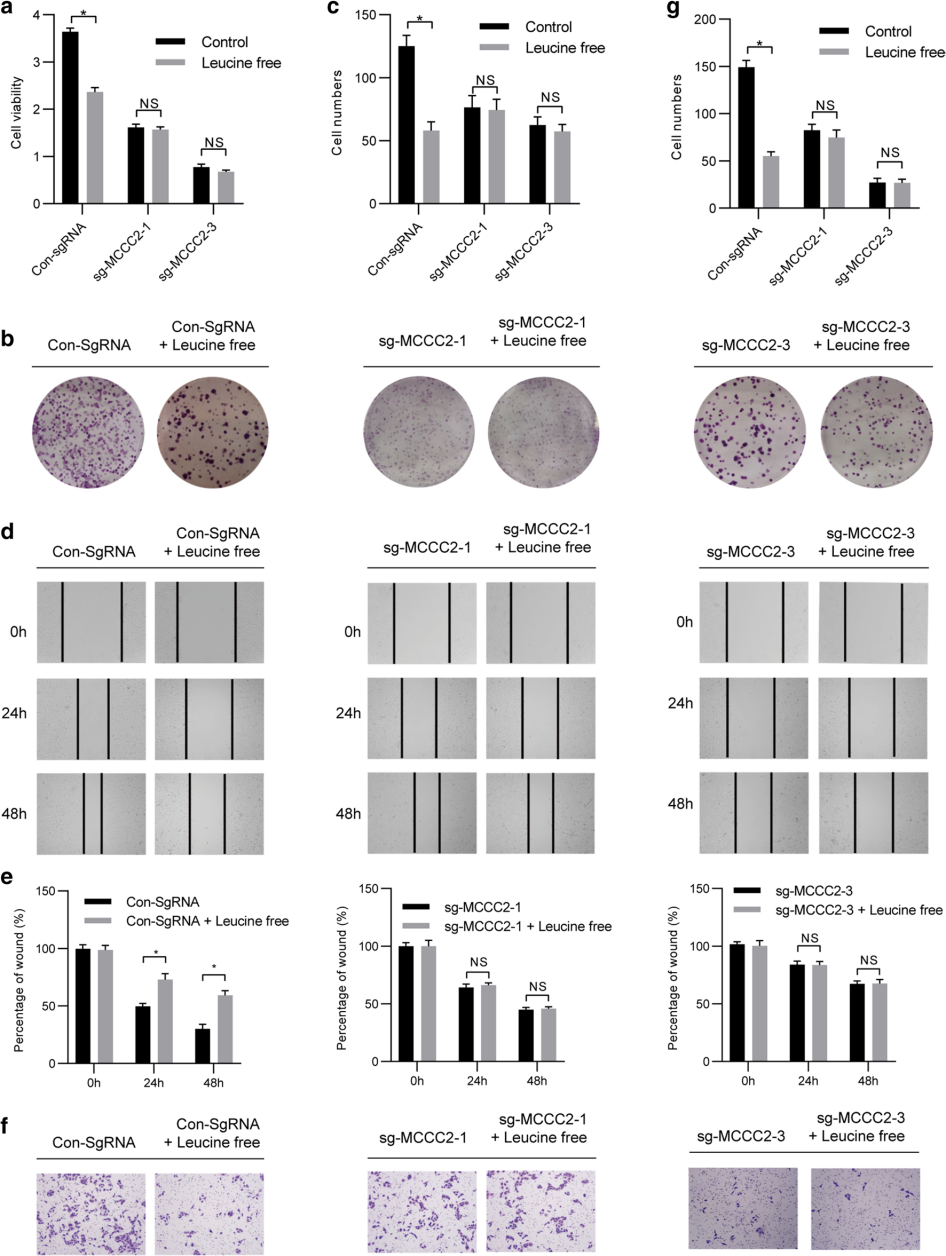
After MCCC2 knockdown, absence of leucine failed to inhibit the biological characteristics of SMMC-7721 cells. **a** Sg-MCCC2 and vector plasmids were stably infected into SMMC-7721 cells. The same number of cells was plated into 96-well plates and cultured under normal or leucine-free^−^ conditions, and the number and viability of the cells were determined every day by CCK-8 assay. **b–g** Colony-formation, Wound-healing, and Transwell assays were performed with SMMC-7721 cells treated as described above

To further examine the dependence of MCCC2 on leucine, we investigated the effect of leucine deprivation in SMMC-7721 cells transfected with sg-MCCC2. Interestingly, we found that the cell viability of SMMC-7721 cells transfected with sgRNA vector greatly decreased upon leucine deprivation. However, there were no differences in SMMC-7721 cells transfected with sg-MCCC2 with or without leucine (Fig. 6a). Similar results were found in the cell proliferation, migration, and invasion abilities of SMMC-7721 cells through colony formation experiments (Fig. 6b, c), scratch-wound healing assay (Fig. 6d, e), and Transwell experiment, respectively (Fig. 6f, g). Taken together, these findings indicate that the effect of leucine deprivation on HCC cells is dependent on MCCC2.

### MCCC2 promotes ERK activation in HCC cells

**Fig. 7:**
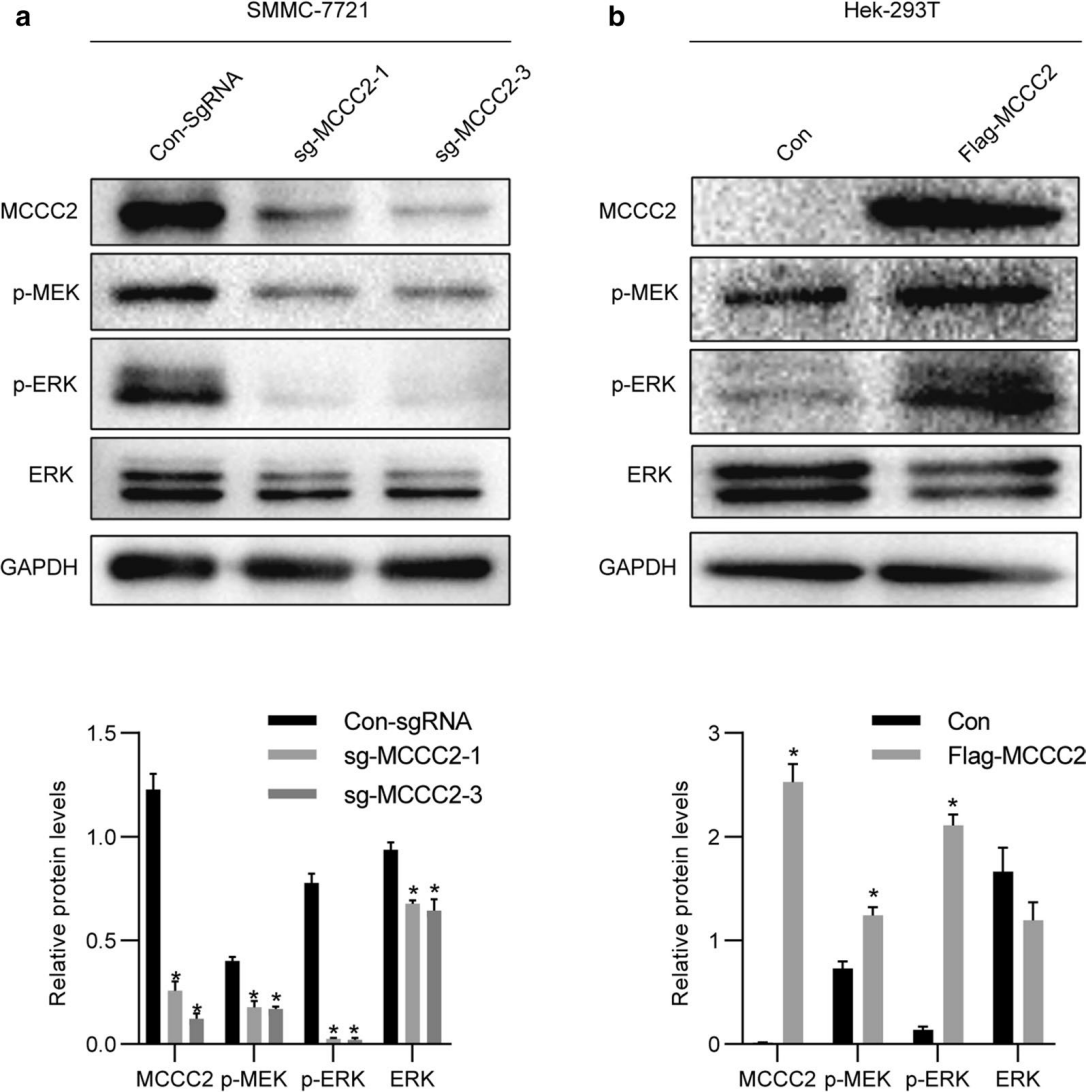
MCCC2 promotes ERK activation. **a** SMMC-7721 cells were transfected with sg-MCCC2 or vector and directly lysed followed by western blot analysis. **b** 293T cells were transfected with Flag-MCCC2 or control and directly lysed followed by western blot analysis

Previous studies have shown that EGF activates the PI3K/AKT pathway and promotes leucine uptake by prostate cancer cells, thereby promoting tumor development [28]. To decipher the underlying mechanisms of MCCC2 in HCC, we also detected the activation of MEK and ERK. We found that the activation of MEK and ERK was greatly reduced in SMMC-7721 cells transfected with sg-MCCC2 (Fig. 7a). Furthermore, overexpression of MCCC2 in Hek-293T cells could induce the activation of MEK and ERK (Fig. 7b). Therefore, our results indicate that MCCC2 can promote ERK activation, which is a common oncogenic pathway frequently observed in cancer cells.

### The profiling of MCCC2 binding proteins and their associated pathways

**Fig. 8:**
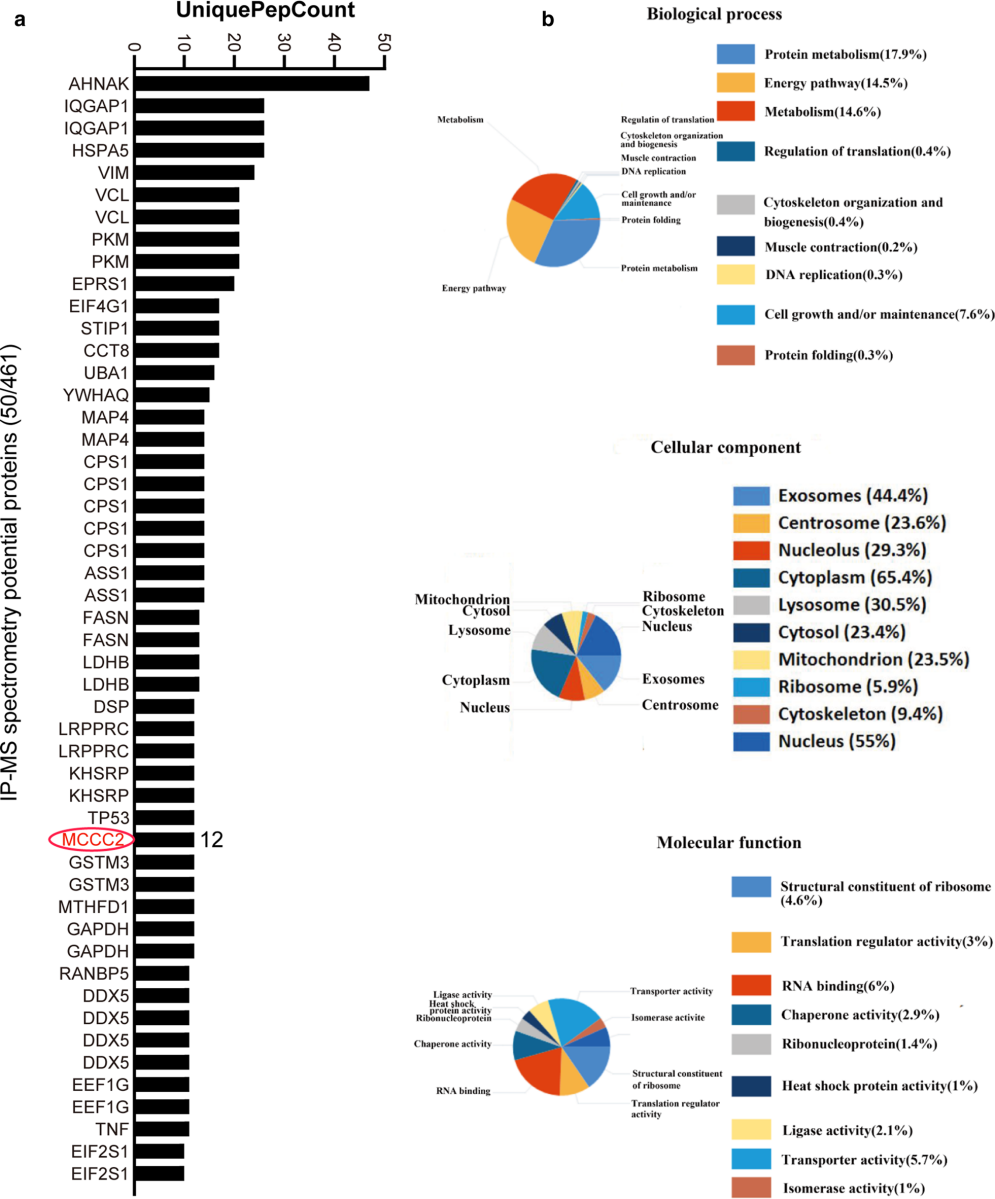
GO analysis of MCCC2-binding proteins. **a** List of proteins from the IP-mass spectrometry data. **b** Detecting MCCC2-binding proteins and predicting biological functions that MCCC2 may participate in by GO analysis

Our results demonstrated a critical role of MCCC2 in HCC cells and the association between MCCC2 and leucine. To fully understand the molecular mechanism and the binding proteins of MCCC2 in HCC cells, an immunoprecipitation assay was carried out using an anti-MCCC2 antibody. Subsequently, mass spectrometry was employed to identify the MCCC2-associated protein (Additional file 1). The top 50 proteins from the IP-mass spectrometry data were listed in Fig. 8a). Through protein mass spectrometry analysis, we identified 461 proteins with at least six unique peptides with high confidence. To further understand the functional significance, the identified proteins were classified according to biological process, molecular function, and cellular component. The main biological processes of these associated proteins are enriched in protein metabolism, energy pathways, and metabolism (Fig. 8b). Therefore, MCCC2 may play an important role in these processes, thereby affecting the occurrence and development of HCC. However, the individual associated proteins identified by IP-MS require further investigation.

## Discussion

At present, the clinical treatment of HCC is largely based on surgical resection and chemotherapy, which has many restrictions and often leads to unsatisfactory results [29]. For the treatment of advanced HCC, only the multi-kinase inhibitor sorafenib has been approved for clinical targeted therapy [30]. Recently, another new multi-kinase inhibitor, regorafenib, and an anti-PD-1 antibody, nivolumab, were established as a new generation of drugs for the treatment of advanced HCC. However, patient prognosis remains poor [31]. Furthermore, the number of potential targets in the preclinical development of HCC therapy is currently very limited. Therefore, there is an urgent need to identify more druggable targets. In this study, we found that MCCC2 plays an important role in HCC. After knocking down MCCC2, the proliferation, migration, and invasion ability of HCC cells significantly decreased. Mechanistically, MCCC2 plays an oncogenic role, at least partially, by regulating the leucine metabolism pathway. Tumor cells often upregulate the uptake of leucine to support cancer development [7, 32]. Besides abnormal uptake of leucine, the enhanced leucine metabolism pathway, such as the upregulation of MCCC2, might also be a critical method of utilizing leucine for cancer growth. Therefore, targeting enzymes involved in leucine metabolism, such as MCCC2, might be a potential method of treating cancer, including HCC.

As an essential amino acid (EA), leucine can only be obtained from diet or microorganisms in mammals [33, 34]. The other 11 amino acids that can be synthesized directly in mammalian cells become non-essential amino acids (NEAAs) [35]. To continue to proliferate, cancer cells require AAs. In the process of transformation, cancer cells develop an abnormal ability to survive and proliferate. These transformed cancer cells are different from normal cells, and in some cases, they rely on specific conditions or specific AAs for survival. In our experimental system, tumor cells were sensitive to leucine deprivation in the external environment (Figs. 5 and 6 and a–d, left column). This observation is consistent with the fact that leucine metabolism is altered in many solid tumors and leucine is needed as a fuel for cancer growth [36]. After knocking down MCCC2, cells become less sensitive to the externally available leucine (Figs. 5 and 6 and a–d, middle and right column). This suggests that cancer cells use key genes to upregulate survival-dependent leucine metabolism. Disruption of these key genes can restore leucine metabolism and make cancer cells less dependent on leucine. Such a mechanism has clinical implications that cancer cells might change drug sensitivity when the oncogenic pathways are changed during cancer development.

We investigated the molecular mechanism by which MCCC2 regulates leucine metabolism as well as other oncogenic functions. To systematically identify the MCCC2-associated proteins, we used IP mass spectrometry to detect potential proteins that interact with MCCC2 in tumor cells. The proteins associated with MCCC2 are enriched in protein metabolism, energy pathways, and metabolism. This is consistent with the function of MCCC2 in leucine metabolism. Among the identified proteins, GCN1 is one of the most interesting. GCN1 is an eIF-2-alpha kinase activator that can activate eIF2α-related pathways. This pathway can affect the utilization of amino acids in colorectal cancer [37]. In addition, the activation of eIF2α caused by the GCN1/GCN20 complex can promote the translation of a large number of proteins [38]. These functions of GCN1 support the observations of this study. It is likely that MCCC2 is also associated with GCN1 and subsequently activates eIF2α-related pathways, thereby affecting the uptake and utilization of amino acids. However, these predictions require experimental validation in future.

In summary, results from in vitro and in vivo experiments support the critical function of MCCC2 in HCC. MCCC2 may regulate leucine metabolism to promote HCC. MCCC2 and its related leucine pathway may be interesting targets for cancer therapy. Therefore, we not only identified a potential target for molecular therapy, but also delineated the important role of leucine in the tumor environment in this study.

## Conclusions

MCCC2 might promote HCC development by supporting leucine oncogenic function.

## Supplementary Information

**Additional flie 1.** The MCCC2-associated proteins identified by IP-MASS spectrometry.

## Data Availability

The datasets used and/or analyzed during the current study are available from the corresponding author upon reasonable request.

## Abbreviations

MCCC2: Methylcrotonoyl-CoA carboxylase 2
HCC: Hepatocellular carcinoma
DMEM: Dulbecco’s modified Eagle’s medium
MCC: 3-Methylcrotonyl-CoA carboxylase
CST: Cell signaling technology
EA: Amino acid
NEAA: Non-essential amino acids
