# Supplementary material for: MCCC2 promotes HCC development by supporting leucine oncogenic function
Source: Cancer Cell Int. 2021 Jan 6;21:22. doi: 10.1186/s12935-020-01722-w (PMC7788835; doi:10.1186/s12935-020-01722-w)
Supplement: Supplementary file 1 — Additional flie 1. The MCCC2-associated proteins identified by IP-MASS spectrometry. [file 12935_2020_1722_MOESM1_ESM.docx]

Table1: The MCCC2-associated proteins identified by IP-MASS spectrometry.

| \| Reference \| UniquePepCount \| \| --- \| --- \| \| AHNAK \| 47 \| \| IQGAP1 \| 26 \| \| IQGAP1 \| 26 \| \| HSPA5 \| 26 \| \| VIM \| 24 \| \| VCL \| 21 \| \| VCL \| 21 \| \| PKM \| 21 \| \| PKM \| 21 \| \| EPRS1 \| 20 \| \| EIF4G1 \| 17 \| \| STIP1 \| 17 \| \| CCT8 \| 17 \| \| UBA1 \| 16 \| \| YWHAQ \| 15 \| \| MAP4 \| 14 \| \| MAP4 \| 14 \| \| CPS1 \| 14 \| \| CPS1 \| 14 \| \| CPS1 \| 14 \| \| CPS1 \| 14 \| \| CPS1 \| 14 \| \| ASS1 \| 14 \| \| ASS1 \| 14 \| \| FASN \| 13 \| \| FASN \| 13 \| \| LDHB \| 13 \| \| LDHB \| 13 \| \| DSP \| 12 \| \| LRPPRC \| 12 \| \| LRPPRC \| 12 \| \| KHSRP \| 12 \| \| KHSRP \| 12 \| \| TP53 \| 12 \| \| MCCC2 \| 12 \| \| GSTM3 \| 12 \| \| GSTM3 \| 12 \| \| MTHFD1 \| 12 \| \| GAPDH \| 12 \| \| GAPDH \| 12 \| \| RANBP5 \| 11 \| \| DDX5 \| 11 \| \| DDX5 \| 11 \| \| DDX5 \| 11 \| \| DDX5 \| 11 \| \| EEF1G \| 11 \| \| EEF1G \| 11 \| \| TNF \| 11 \| \| EIF2S1 \| 10 \| \| EIF2S1 \| 10 \| \| PA2G4 \| 10 \| \| TRIM28 \| 10 \| \| CAP1 \| 10 \| \| CAP1 \| 10 \| \| CAP1 \| 10 \| \| SLC3A2 \| 10 \| \| INS \| 10 \| \| ATP5F1B \| 10 \| \| ATP5B \| 10 \| \| ALDOA \| 10 \| \| ALDOA \| 10 \| \| ALDOA \| 10 \| \| SDHA \| 10 \| \| SDHA \| 10 \| \| SDHA \| 10 \| \| AHCY \| 10 \| \| AHCY \| 10 \| \| EIF4A1 \| 10 \| \| EIF4A1 \| 10 \| \| PRDX1 \| 10 \| \| ATP1A1 \| 9 \| \| ATP1A1 \| 9 \| \| DDX3X \| 9 \| \| EEF1D \| 9 \| \| EEF1D \| 9 \| \| EEF1D \| 9 \| \| EEF1D \| 9 \| \| YWHAE \| 9 \| \| RPS4X \| 9 \| \| RACK1 \| 9 \| \| CKAP4 \| 9 \| \| DDX17 \| 9 \| \| DDX17 \| 9 \| \| DDX17 \| 9 \| \| DDX17 \| 9 \| \| FUBP1 \| 9 \| \| FUBP1 \| 9 \| \| FUBP1 \| 9 \| \| FUBP1 \| 9 \| \| FUBP1 \| 9 \| \| TNF \| 9 \| \| GPI \| 9 \| \| GPI \| 9 \| \| GPI \| 9 \| \| GPI \| 9 \| \| GPI \| 9 \| \| CFTR \| 9 \| \| RPL13A \| 9 \| \| RPL13A \| 9 \| \| RPL13A \| 9 \| \| EIF3B \| 9 \| \| EIF3S9 \| 9 \| \| EIF3S9 \| 9 \| \| EIF3B \| 9 \| \| HNRPR \| 9 \| \| HNRPR \| 9 \| \| HNRPR \| 9 \| \| HNRPR \| 9 \| \| RPLP0 \| 9 \| \| DKFZp686J01190 \| 9 \| \| HNRPC \| 9 \| \| HNRPC \| 9 \| \| HNRNPC \| 9 \| \| HNRNPC \| 9 \| \| HNRNPC \| 9 \| \| ANXA1 \| 9 \| \| ANXA1 \| 9 \| \| HNRNPA2B1 \| 9 \| \| RDX \| 9 \| \| RDX \| 9 \| \| RDX \| 9 \| \| RDX \| 9 \| \| TUBB3 \| 9 \| \| TUBB3 \| 9 \| \| TUBB3 \| 9 \| \| TUBB3 \| 9 \| \| TUBB3 \| 9 \| \| NPM1 \| 9 \| \| NPM1 \| 9 \| \| NPM1 \| 9 \| \| EEF1A2 \| 9 \| \| EEF1A2 \| 9 \| \| HSPA1L \| 9 \| \| HSPA1L \| 9 \| \| HSPA1L \| 9 \| \| HSPA1L \| 9 \| \| HSPA1L \| 9 \| \| HSPA1L \| 9 \| \| HSPA1L \| 9 \| \| VCP \| 8 \| \| RPS6 \| 8 \| \| RPS6 \| 8 \| \| RPS6 \| 8 \| \| RPS6 \| 8 \| \| CCT2 \| 8 \| \| TCP1 \| 8 \| \| PFKP \| 8 \| \| FKBP4 \| 8 \| \| SRSF1 \| 8 \| \| SRSF1 \| 8 \| \| NSUN2 \| 8 \| \| KPNB1 \| 8 \| \| KPNB1 \| 8 \| \| GARS \| 8 \| \| GARS \| 8 \| \| TCOF1 \| 8 \| \| TCOF1 \| 8 \| \| TCOF1 \| 8 \| \| TCOF1 \| 8 \| \| APOE \| 8 \| \| TPM1 \| 8 \| \| TPM1 \| 8 \| \| TPM1 \| 8 \| \| TPM1 \| 8 \| \| TPM1 \| 8 \| \| TPM1 \| 8 \| \| TPM1 \| 8 \| \| TPM1 \| 8 \| \| GART \| 8 \| \| GART \| 8 \| \| GART \| 8 \| \| CAD \| 8 \| \| CAD \| 8 \| \| SFN \| 8 \| \| RPS3A \| 8 \| \| RPS3A \| 8 \| \| RPS3A \| 8 \| \| RPS3A \| 8 \| \| CAPZB \| 8 \| \| CAPZB \| 8 \| \| CAPZB \| 8 \| \| CAPZB \| 8 \| \| CAPZB \| 8 \| \| PABPC4 \| 8 \| \| SLC25A5 \| 8 \| \| SLC25A5 \| 8 \| \| RBBP4 \| 8 \| \| EIF3C \| 8 \| \| EIF3C \| 8 \| \| EIF3C \| 8 \| \| EIF3C \| 8 \| \| EIF3C \| 8 \| \| EIF3S8 \| 8 \| \| EIF3CL \| 8 \| \| EIF3C \| 8 \| \| EIF3C \| 8 \| \| YWHAB \| 8 \| \| YWHAB \| 8 \| \| HNRNPA1 \| 8 \| \| HNRPA1 \| 8 \| \| HNRPA1 \| 8 \| \| HNRPA1 \| 8 \| \| HNRNPA1 \| 8 \| \| TRAP1 \| 8 \| \| TRAP1 \| 8 \| \| TRAP1 \| 8 \| \| TRAP1 \| 8 \| \| TRAP1 \| 8 \| \| TPI1 \| 8 \| \| TPI1 \| 8 \| \| CFL1 \| 8 \| \| CFL1 \| 8 \| \| CFL1 \| 8 \| \| CFL1 \| 8 \| \| PPIA \| 8 \| \| PPIA \| 8 \| \| PPIA \| 8 \| \| PPIA \| 8 \| \| PHGDH \| 7 \| \| PHGDH \| 7 \| \| PHGDH \| 7 \| \| ITGB1 \| 7 \| \| CTPS1 \| 7 \| \| CTPS1 \| 7 \| \| EIF2S3 \| 7 \| \| TUFM \| 7 \| \| TUFM \| 7 \| \| RANBP2 \| 7 \| \| RPS11 \| 7 \| \| HRNR \| 7 \| \| DDX21 \| 7 \| \| NUDC \| 7 \| \| TLN1 \| 7 \| \| TLN1 \| 7 \| \| YWHAH \| 7 \| \| YWHAH \| 7 \| \| FKBP10 \| 7 \| \| FKBP10 \| 7 \| \| BZW2 \| 7 \| \| BZW2 \| 7 \| \| BZW2 \| 7 \| \| BZW2 \| 7 \| \| BZW2 \| 7 \| \| BZW2 \| 7 \| \| ATP2A2 \| 7 \| \| ATP2A2 \| 7 \| \| RBM14 \| 7 \| \| RBM14 \| 7 \| \| PLIN3 \| 7 \| \| PLIN3 \| 7 \| \| COPA \| 7 \| \| COPA \| 7 \| \| COPA \| 7 \| \| COPA \| 7 \| \| THRAP3 \| 7 \| \| THRAP3 \| 7 \| \| ARCN1 \| 7 \| \| ARCN1 \| 7 \| \| ARCN1 \| 7 \| \| ARCN1 \| 7 \| \| MCM3 \| 7 \| \| MCM3 \| 7 \| \| MCM3 \| 7 \| \| CALU \| 7 \| \| CALU \| 7 \| \| CALU \| 7 \| \| CALU \| 7 \| \| SMC4 \| 7 \| \| SMC4 \| 7 \| \| SMC4 \| 7 \| \| SMC4 \| 7 \| \| HADHA \| 7 \| \| HADHA \| 7 \| \| HADHA \| 7 \| \| RPN1 \| 7 \| \| RPN1 \| 7 \| \| RPN1 \| 7 \| \| RPN1 \| 7 \| \| RPN1 \| 7 \| \| TCP1 \| 7 \| \| TCP1 \| 7 \| \| CCT6A \| 7 \| \| CCT6A \| 7 \| \| KIF5B \| 7 \| \| KIF5B \| 7 \| \| KIF5B \| 7 \| \| KIF5B \| 7 \| \| ANXA4 \| 7 \| \| ANXA4 \| 7 \| \| ANXA4 \| 7 \| \| ANXA4 \| 7 \| \| ANXA4 \| 7 \| \| GANAB \| 7 \| \| GANAB \| 7 \| \| RPL35A \| 7 \| \| PPIB \| 7 \| \| PPIB \| 7 \| \| RPL12 \| 7 \| \| RPS19 \| 7 \| \| RPS19 \| 7 \| \| MATR3 \| 7 \| \| MATR3 \| 7 \| \| MATR3 \| 7 \| \| MATR3 \| 7 \| \| PABPC1 \| 7 \| \| PABPC1 \| 7 \| \| PABPC1 \| 7 \| \| PABPC1 \| 7 \| \| PABPC1 \| 7 \| \| EIF4B \| 7 \| \| EIF4B \| 7 \| \| EIF4B \| 7 \| \| EIF4B \| 7 \| \| SLC25A6 \| 7 \| \| SLC25A6 \| 7 \| \| SLC25A6 \| 7 \| \| PRDX6 \| 7 \| \| PRDX6 \| 7 \| \| SYNCRIP \| 7 \| \| SYNCRIP \| 7 \| \| RPSA \| 7 \| \| RPSA \| 7 \| \| RPSA \| 7 \| \| RPSA \| 7 \| \| RPL26 \| 7 \| \| RPL26 \| 7 \| \| RBBP7 \| 7 \| \| RBBP7 \| 7 \| \| RBBP7 \| 7 \| \| PFN1 \| 7 \| \| HSPA6 \| 7 \| \| HSPA6 \| 7 \| \| HSPA6 \| 7 \| \| HSPA6 \| 7 \| \| HSPA6 \| 7 \| \| GAPD \| 7 \| \| RPS27A \| 7 \| \| RPS27A \| 7 \| \| HEL112 \| 7 \| \| HEL112 \| 6 \| \| UGDH \| 6 \| \| HMGB1 \| 6 \| \| HMGB1 \| 6 \| \| ANXA3 \| 6 \| \| ANXA3 \| 6 \| \| XRCC5 \| 6 \| \| XRCC5 \| 6 \| \| PRKCSH \| 6 \| \| PRKCSH \| 6 \| \| PRKCSH \| 6 \| \| PRKCSH \| 6 \| \| PSMA1 \| 6 \| \| PSMA1 \| 6 \| \| PSMA1 \| 6 \| \| COPB2 \| 6 \| \| VDAC2 \| 6 \| \| VDAC2 \| 6 \| \| VDAC2 \| 6 \| \| VDAC2 \| 6 \| \| PSMD2 \| 6 \| \| PSMD2 \| 6 \| \| SMC1A \| 6 \| \| SMC1L1 \| 6 \| \| DDB1 \| 6 \| \| TXNDC5 \| 6 \| \| DKFZp666I134 \| 6 \| \| STRF8 \| 6 \| \| TXNDC5 \| 6 \| \| GCN1 \| 6 \| \| PRIC295 \| 6 \| \| HDLBP \| 6 \| \| HDLBP \| 6 \| \| HDLBP \| 6 \| \| FTH1 \| 6 \| \| FTH1 \| 6 \| \| FTH1 \| 6 \| \| FTH1 \| 6 \| \| FTH1 \| 6 \| \| GOT2 \| 6 \| \| GOT2 \| 6 \| \| GOT2 \| 6 \| \| FH \| 6 \| \| FH \| 6 \| \| FH \| 6 \| \| CD44 \| 6 \| \| CD44 \| 6 \| \| CD44 \| 6 \| \| CD44 \| 6 \| \| CD44 \| 6 \| \| CD44 \| 6 \| \| ILF3 \| 6 \| \| ILF3 \| 6 \| \| ILF3 \| 6 \| \| ILF3 \| 6 \| \| ILF3 \| 6 \| \| ILF3 \| 6 \| \| SMC3 \| 6 \| \| SMC3 \| 6 \| \| SMC3 \| 6 \| \| MCCC1 \| 6 \| \| GLUD1 \| 6 \| \| GLUD1 \| 6 \| \| GLUD1 \| 6 \| \| GLUD1 \| 6 \| \| GLUD1 \| 6 \| \| GLUD1 \| 6 \| \| GLUD1 \| 6 \| \| ST13 \| 6 \| \| ST13 \| 6 \| \| ST13 \| 6 \| \| RRM1 \| 6 \| \| RRM1 \| 6 \| \| RRM1 \| 6 \| \| RAB1A \| 6 \| \| RAB1A \| 6 \| \| SNRNP200 \| 6 \| \| PAICS \| 6 \| \| PAICS \| 6 \| \| PAICS \| 6 \| \| PAICS \| 6 \| \| ANP32B \| 6 \| \| ANP32B \| 6 \| \| DDX48 \| 6 \| \| EIF4A3 \| 6 \| \| CBX3 \| 6 \| \| CBX3 \| 6 \| \| CBX3 \| 6 \| \| PSMC3 \| 6 \| \| PSMC3 \| 6 \| \| PSMC3 \| 6 \| \| ALB \| 6 \| \| ALB \| 6 \| \| ALB \| 6 \| \| ALB \| 6 \| \| ALB \| 6 \| \| ALB \| 6 \| \| VDAC1 \| 6 \| \| VDAC1 \| 6 \| \| VDAC1 \| 6 \| \| EIF2S2 \| 6 \| \| EIF2S2 \| 6 \| \| EIF2S2 \| 6 \| \| SRSF2 \| 6 \| \| SRSF2 \| 6 \| \| SRSF2 \| 6 \| \| SRSF2 \| 6 \| \| SRSF2 \| 6 \| \| RAB1B \| 6 \| \| RAB1B \| 6 \| \| CALR \| 6 \| \| CALR \| 6 \| \| CALR \| 6 \| \| YBX1 \| 6 \| \| PRDX4 \| 6 \| \| PRDX4 \| 6 \| \| GSTP1 \| 6 \| \| GSTP1 \| 6 \| \| GSTP1 \| 6 \| \| GSTP1 \| 6 \| \| HSP90AA2P \| 6 \| \| HSP90AA2P \| 6 \| |  |
| --- | --- | --- | --- | --- | --- | --- | --- | --- | --- | --- | --- | --- | --- | --- | --- | --- | --- | --- | --- | --- | --- | --- | --- | --- | --- | --- | --- | --- | --- | --- | --- | --- | --- | --- | --- | --- | --- | --- | --- | --- | --- | --- | --- | --- | --- | --- | --- | --- | --- | --- | --- | --- | --- | --- | --- | --- | --- | --- | --- | --- | --- | --- | --- | --- | --- | --- | --- | --- | --- | --- | --- | --- | --- | --- | --- | --- | --- | --- | --- | --- | --- | --- | --- | --- | --- | --- | --- | --- | --- | --- | --- | --- | --- | --- | --- | --- | --- | --- | --- | --- | --- | --- | --- | --- | --- | --- | --- | --- | --- | --- | --- | --- | --- | --- | --- | --- | --- | --- | --- | --- | --- | --- | --- | --- | --- | --- | --- | --- | --- | --- | --- | --- | --- | --- | --- | --- | --- | --- | --- | --- | --- | --- | --- | --- | --- | --- | --- | --- | --- | --- | --- | --- | --- | --- | --- | --- | --- | --- | --- | --- | --- | --- | --- | --- | --- | --- | --- | --- | --- | --- | --- | --- | --- | --- | --- | --- | --- | --- | --- | --- | --- | --- | --- | --- | --- | --- | --- | --- | --- | --- | --- | --- | --- | --- | --- | --- | --- | --- | --- | --- | --- | --- | --- | --- | --- | --- | --- | --- | --- | --- | --- | --- | --- | --- | --- | --- | --- | --- | --- | --- | --- | --- | --- | --- | --- | --- | --- | --- | --- | --- | --- | --- | --- | --- | --- | --- | --- | --- | --- | --- | --- | --- | --- | --- | --- | --- | --- | --- | --- | --- | --- | --- | --- | --- | --- | --- | --- | --- | --- | --- | --- | --- | --- | --- | --- | --- | --- | --- | --- | --- | --- | --- | --- | --- | --- | --- | --- | --- | --- | --- | --- | --- | --- | --- | --- | --- | --- | --- | --- | --- | --- | --- | --- | --- | --- | --- | --- | --- | --- | --- | --- | --- | --- | --- | --- | --- | --- | --- | --- | --- | --- | --- | --- | --- | --- | --- | --- | --- | --- | --- | --- | --- | --- | --- | --- | --- | --- | --- | --- | --- | --- | --- | --- | --- | --- | --- | --- | --- | --- | --- | --- | --- | --- | --- | --- | --- | --- | --- | --- | --- | --- | --- | --- | --- | --- | --- | --- | --- | --- | --- | --- | --- | --- | --- | --- | --- | --- | --- | --- | --- | --- | --- | --- | --- | --- | --- | --- | --- | --- | --- | --- | --- | --- | --- | --- | --- | --- | --- | --- | --- | --- | --- | --- | --- | --- | --- | --- | --- | --- | --- | --- | --- | --- | --- | --- | --- | --- | --- | --- | --- | --- | --- | --- | --- | --- | --- | --- | --- | --- | --- | --- | --- | --- | --- | --- | --- | --- | --- | --- | --- | --- | --- | --- | --- | --- | --- | --- | --- | --- | --- | --- | --- | --- | --- | --- | --- | --- | --- | --- | --- | --- | --- | --- | --- | --- | --- | --- | --- | --- | --- | --- | --- | --- | --- | --- | --- | --- | --- | --- | --- | --- | --- | --- | --- | --- | --- | --- | --- | --- | --- | --- | --- | --- | --- | --- | --- | --- | --- | --- | --- | --- | --- | --- | --- | --- | --- | --- | --- | --- | --- | --- | --- | --- | --- | --- | --- | --- | --- | --- | --- | --- | --- | --- | --- | --- | --- | --- | --- | --- | --- | --- | --- | --- | --- | --- | --- | --- | --- | --- | --- | --- | --- | --- | --- | --- | --- | --- | --- | --- | --- | --- | --- | --- | --- | --- | --- | --- | --- | --- | --- | --- | --- | --- | --- | --- | --- | --- | --- | --- | --- | --- | --- | --- | --- | --- | --- | --- | --- | --- | --- | --- | --- | --- | --- | --- | --- | --- | --- | --- | --- | --- | --- | --- | --- | --- | --- | --- | --- | --- | --- | --- | --- | --- | --- | --- | --- | --- | --- | --- | --- | --- | --- | --- | --- | --- | --- | --- | --- | --- | --- | --- | --- | --- | --- | --- | --- | --- | --- | --- | --- | --- | --- | --- | --- | --- | --- | --- | --- | --- | --- | --- | --- | --- | --- | --- | --- | --- | --- | --- | --- | --- | --- | --- | --- | --- | --- | --- | --- | --- | --- | --- | --- | --- | --- | --- | --- | --- | --- | --- | --- | --- | --- | --- | --- | --- | --- | --- | --- | --- | --- | --- | --- | --- | --- | --- | --- | --- | --- | --- | --- | --- | --- | --- | --- | --- | --- | --- | --- | --- | --- | --- | --- | --- | --- | --- | --- | --- | --- | --- | --- | --- | --- | --- | --- | --- | --- | --- | --- | --- | --- | --- | --- | --- | --- | --- | --- | --- | --- | --- | --- | --- | --- | --- | --- | --- | --- | --- | --- | --- | --- | --- | --- | --- | --- | --- | --- | --- | --- | --- | --- | --- | --- | --- | --- | --- | --- | --- | --- | --- | --- | --- | --- | --- | --- | --- | --- | --- | --- | --- | --- | --- | --- | --- | --- | --- | --- | --- | --- | --- | --- | --- | --- | --- | --- | --- | --- | --- | --- | --- | --- | --- | --- | --- | --- | --- | --- | --- | --- | --- | --- | --- | --- | --- | --- | --- | --- | --- | --- | --- | --- | --- | --- | --- | --- | --- | --- | --- | --- | --- | --- | --- | --- | --- | --- | --- | --- | --- | --- | --- | --- | --- | --- | --- | --- | --- | --- | --- | --- | --- | --- | --- | --- | --- | --- | --- | --- | --- | --- | --- | --- | --- | --- | --- | --- | --- | --- | --- | --- | --- | --- | --- | --- | --- | --- | --- | --- | --- | --- | --- | --- | --- | --- | --- | --- | --- | --- | --- | --- | --- | --- | --- | --- | --- | --- | --- | --- | --- | --- | --- | --- | --- | --- | --- | --- | --- | --- | --- | --- | --- | --- | --- | --- | --- | --- | --- | --- | --- | --- | --- | --- | --- | --- | --- | --- | --- | --- | --- | --- | --- | --- | --- | --- | --- | --- | --- | --- | --- | --- | --- | --- | --- | --- | --- | --- | --- |
|  |  |
|  |  |
|  |  |
|  |  |
|  |  |
|  |  |
|  |  |
|  |  |
|  |  |
|  |  |
|  |  |
|  |  |
|  |  |
|  |  |
|  |  |
|  |  |
|  |  |
|  |  |
|  |  |
|  |  |
|  |  |
|  |  |
